# ﻿Begoniafimbristipulasubsp.siamensis (sect. Diploclinium, Begoniaceae), a new taxon of the megadiverse genus endemic to Thailand

**DOI:** 10.3897/phytokeys.218.85699

**Published:** 2023-01-10

**Authors:** Sirilak Radbouchoom, Thamarat Phutthai, Harald Schneider

**Affiliations:** 1 Center for Integrative Conservation, Xishuangbanna Tropical Botanical Garden, Chinese Academy of Sciences, Menglun 666303, Yunnan, China Center for Integrative Conservation, Xishuangbanna Tropical Botanical Garden, Chinese Academy of Sciences Menglun China; 2 University of Chinese Academy of Sciences, Beijing 100049, China University of Chinese Academy of Sciences Beijing China; 3 Faculty of Environment and Resource Studies, Mahidol University (Salaya Campus), Nakhon Pathom 73170, Thailand Mahidol University Nakhon Pathom Thailand

**Keywords:** China, endemic, new subspecies, taxonomy, Thailand

## Abstract

The genus *Begonia* has not only been recognised to be one of the mega-diverse plant genera but also as one found to comprise many undiscovered species. In particular, the increase of extensive field surveys in tropical regions of Southeast Asia has added to the discovery of many new species that are often found only in a few localities. In this study, the new taxon Begoniafimbristipulasubsp.siamensis**subsp. nov.** from Thailand is described. The Thailand accessions are highly similar in their morphology to accessions of *B.fimbristipula* from southern China but differ in their tuber shape, peduncle trichomes, petiole trichomes and number of female tepals. The new taxon has been found only in the northern parts of Thailand occurring at elevations above 1,300 meters. The new findings not only contribute to our knowledge of the plant diversity of Thailand but provide also critical information contributing to the protection of this species. In China, this species is endangered which is of special concern given its utilisation as a medical herb in traditional Chinese medicine. Considering IUCN Red List Categories, the new subspecies is considered to be Vulnerable. The disjunct distribution of the two subspecies of *B.fimbristipula* encourages urgently needed comparative taxonomic studies across the Indo-Burma biodiversity hotspot.

## ﻿Introduction

With about 2,000 accepted species, the pantropical, herbaceous flowering plant genus *Begonia* L. (Linnaeus 1753) (Begoniaceae, Cucurbitales) is an outstanding example of a mega-diverse plant genus (Stevens 2001; Hughes et al. 2015; Li et al. 2022). In recent years, the number of known species has rapidly expanded as a consequence of a combination of factors including extensive field surveys in tropical regions (Hughes et al. 2015; Moonlight et al. 2018). Species surveys are especially needed to document the diversity in subtropical to tropical Asia because this region, with 1,159 recorded species, is a diversity hotspot of *Begonia* (Hughes et al. 2015). Taxonomic studies of this mega-diverse genus are challenged by several aspects including (1) the vast number of species required to be compared, (2) the high frequency of disjunct local occurrences, and (3) phenotypic plasticity observed in many species (McLellan 2000; Wang et al. 2016; Wahlsteen 2021). Until now, 58 species of *Begonia* have been recorded as occurring in Thailand of which about 40 percent are endemic (Phutthai et al. 2019). Here, we report results of our ongoing efforts to improve the documentation of this mega-diverse genus in Thailand. These research activities have been motivated by two key-assumptions. Firstly, the diversity of *Begonia* in Thailand is incompletely known and thus several species await to be found. Secondly, at least some, if not many, species of Thailand’s *Begonia* are threatened as a consequence of the ongoing biodiversity crisis caused by anthropogenic activities.

In our studies, we focused specifically on occurrences of BegoniasectionDiploclinium (Lindl.) A.DC. (Lindley 1847; de Candolle 1859). This section requires a re-definition because the currently used circumscription has been considered to be a taxonomic dustbin (Shui et al. 2002; Rubite et al. 2013). This expectation has been confirmed in phylogenetic studies recovering the section to be polyphyletic (Thomas et al. 2011; Rubite et al. 2013). As a consequence, all species of this section occurring in the Philippines were reassigned to Begoniasect.Baryandra (Rubite et al. 2013). 19 out of the 58 *Begonia* species occurring in Thailand have been assigned to Begoniasect.Diploclinium (Phutthai et al. 2019). These placements have not yet been confirmed using phylogenetic studies and the current definition of the section is somewhat ambiguous, in that it is a combination of two key characters for the section, namely tuberous habit and bifid placentae (Doorenbos et al. 1998). Besides the challenges of classification of recognised species, many species of this section are expected to still await their discovery. The main aim of our study is to estimate the real number of species belonging to Begoniasect.Diploclinium that occur in Thailand. As a first step toward achieving this goal, field surveys were carried out in remote parts of Thailand to collect accessions of putative unknown taxa. These accessions were then carefully compared to previously described species by considering not only species known to occur in Thailand but also species occurring in other parts of continental Asia including southern China.

## ﻿Methods

New accessions were examined and photographed in their natural habitats during field surveys in northern and north-eastern Thailand. They were consequently carefully compared to previously described species and as far as possible voucher specimens either by access to the voucher itself or to images. Special attention was given to type vouchers. To avoid describing an already published species, extensive comparisons were carried out with known species that showed high similarity in our first round of examination. The newly obtained voucher specimens were deposited at the two key herbaria in Thailand namely Forest Herbarium at Bangkok (BKF) and Queen Sirikit Botanic Garden Herbarium at Chiang Mai (QBG) besides several prominent international herbaria (K, E, P, HAST). Distribution maps were generated using QGIS software (QGIS 2021) by combining the records obtained during our field surveys with records from specimens deposited in herbaria. The assessment of the International Union for Conservation of Nature (IUCN) Red List status was evaluated using information from field observation and the available specimen records.

## ﻿Results

The newly recognized taxon has a tuberous habit, cymose bisexual inflorescences with four male tepals and three to five-female tepals, trilocular fruit, and axillary bifid placentation. This character combination is found exclusively in Begoniasect.Diploclinium. The new taxon resembles *B.fimbristipula* Hance (Hance 1883), not previously recorded in Thailand. Comparisons of living plants and herbarium specimens recovered morphological characters that distinguished the accessions from Thailand from accessions of that species collected in southern China (Table 1). The distribution in Thailand and southern China shows a disjunct range with no records reported for eastern Myanmar, northern Laos and northern Vietnam (Fig. 2).

**Table 1.** Comparison of diagnostic morphological characteristics among BegoniafimbristipulaHancesubsp.siamensis Phutthai & S.Radbouchoom, B.fimbristipulaHancesubsp.fimbristipula and *B.poilanei* Kiew.

	B.fimbristipulasubsp.siamensis	B.fimbristipulasubsp.fimbristipula	*B.poilanei*
**Stem**	globose or sub-elongate tubers	globose or sub-globose tubers	elongate tubers
**Petiole**	densely red villous and pubescent	villous	densely hairy
**Leaf blade abaxially**	densely villous	sparsely pubescent and densely villous to red villous	sparsely pubescent
**Peduncle**	sparsely red villous	glabrous	hairy
**Tepal number in pistillate flowers**	5	3	3
**Placentation**	axile	axile	septal
**Distribution**	Northern and North-eastern Thailand (Chiangmai, Nan, Phitsanulok)	Southern China (Fujian, Guangdong, Guangxi, Hainan, Hunan, Jiangxi, Zhejiang)	China (Hainan); Vietnam (Haut Donai district)

### ﻿Taxonomic treatment

#### ﻿ Begonia fimbristipula Hance subsp. siamensis

Taxon classificationPlantaeCucurbitalesBegoniaceae

Phutthai & S.Radbouchoom subsp. nov.

FCAC9DE4-77F2-5DDE-9500-C099FB26FC5C

urn:lsid:ipni.org:names:77311666-1

Figs 1 , 2

##### Type.

Thailand • Phitsanulok Province, Nakornthai, Phu Hin Rong Kla national park, Lan Hin Pum rout; 1300 m a.s.l.; 29 Oct. 2001; S. Watthana, P. Suksathan 1570; herb on sand stone rather shed and wet; (holotype QBG! 21778, isotype BKF! SN187939).

##### Description.

Perennial monoecious acaulescent herb, 10–25 cm tall. ***Tuber*** globose or sub-elongate 0.7–2.0 cm long, with fibrous roots. ***Leaves*** 1–3; petiole maroon, 9.0–13.5 cm long, densely red villous and pubescent; lamina basifixed, succulent, broadly ovate, symmetric or slightly asymmetric, adaxial surface green, sometimes sparsely white spotted, densely villous, conspicuous dark green band 2–3 mm wide along the midrib, secondary and tertiary veins, abaxial surface green or red, densely red villous, conspicuous maroon band 2–3 mm wide along the secondary and tertiary veins, 6.5–15×4.6–12 cm, base cordate, margin denticulate and ciliate, apex acuminate, venation palmate-pinnate, primary veins 6–7, secondary veins branching dichotomous 2–4, prominent beneath. ***Stipules***, ovate-triangular, apex acute, margin ciliate, 2.0–3.0×1.2–1.7 mm, red villous. ***Inflorescences*** arising directly from tuber, bisexual, cymose, branching 1–3 times; peduncles maroon, 11.5–25 cm long, sparsely red villous; bracts soon falling, membranaceous, oblong, glabrous, apex obtuse, margin fimbriae. ***Staminate flowers***: pedicel maroon, 1.5–2.0 cm long, glabrous, erect; tepals 4, white-pinkish to pink with the pale pink flash at the centre, glabrous both sides; outer 2 ovate to suborbicular, 0.8–1.0×0.3–0.5 cm, base rounded to cuneate, margin entire, apex obtuse to rounded; inner 2 narrowly elliptic or lanceolate-elliptic, 0.7–1.2×0.7–1.0 cm, base cuneate, margin entire, apex obtuse to rounded; androecium zygomorphic; stamens 10–25, yellow; filaments fused at base, c.1.0 mm long; anthers clavate, apex rounded, 0.3–1.0 mm long, basifixed, dehiscing by 2 short slits. ***Pistillate flowers***: pedicel maroon, 1.5–2.0 cm long, tepals 5, white-pinkish to pink with a pale pink flash at the centre, glabrous both sides; outer 3, ovate-broadly ovate, 0.7–1×0.3–0.4 cm, apex obtuse to rounded, margin entire, base cuneate; inner 2, ovate to lanceolate-elliptic, 0.6–0.9×0.3–0.6 cm, apex obtuse to rounded, margin entire, base cuneate; styles 3, yellow, fused at base 1.0–2.0 mm long, bilobed, crescent-shaped, spirally twisted; ovary with 3 unequal wings, oblong, 0.5–1.0×0.4–0.6 cm, glabrous; placentation axile, placentae bilamellate, locules 3. ***Fruit*** oblong, unequally 3 winged, capsule, nodding, pink to pale green, 1.0–2.2×0.9–1.1 cm included wings, abaxial wing falcate or elliptic, extended 1.0–1.3×0.7–0.9 cm, wider than lateral wings, lateral wings, narrowly triangular, 0.7–1×0.2–0.3 cm.

##### Habitat.

**Figure 1.**
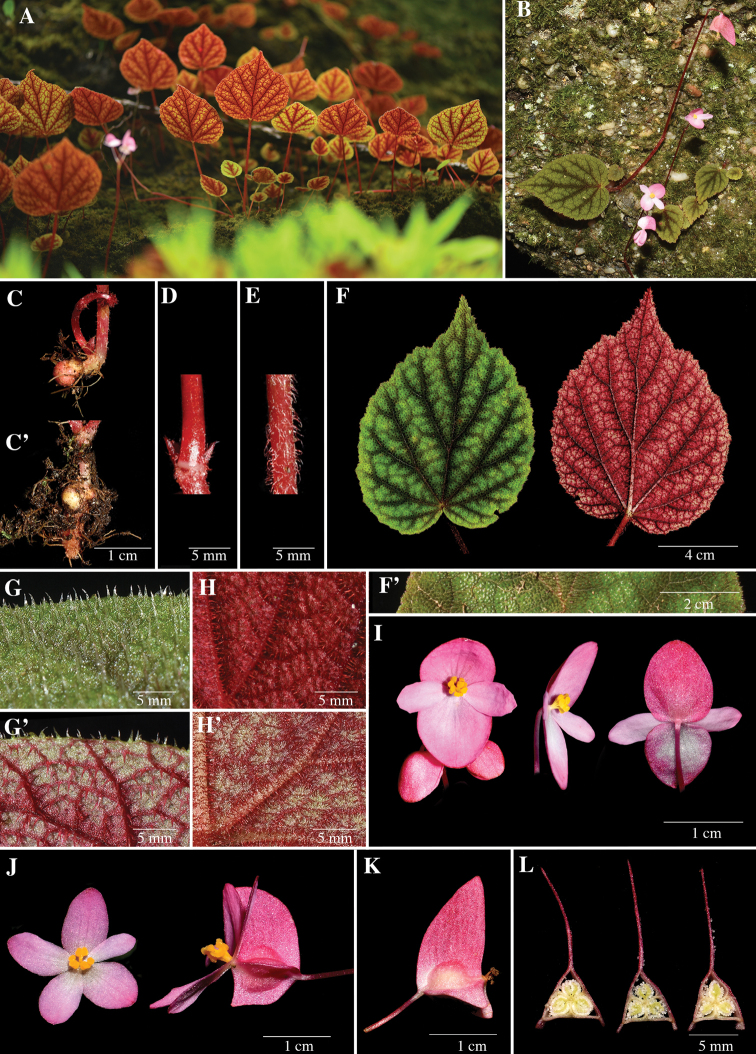
BegoniafimbristipulaHancesubsp.siamensis Phutthai & S.Radbouchoom subsp. nov. **A** habitat **B** habit **C** tuber globose **C**’ tuber sub-elongate **D** peduncle **E** petiole **F** leaf adaxial and abaxial surface **F**’ close-up of white spots on adaxial surface of leaf **G** close-up of trichome on adaxial surface of greenish leaf **G**’ close-up of trichome on abaxial surface of leaf **H** close-up of abaxial surface of reddish leaf **H**’ close-up of abaxial surface of reddish leaf **I** staminate flower visualised using face view, side view, back view **J** pistillate flower visualised using face view, side view **K** fruit **L** cross sections of young ovary in the sequence top, middle and bottom.

**Figure 2.**
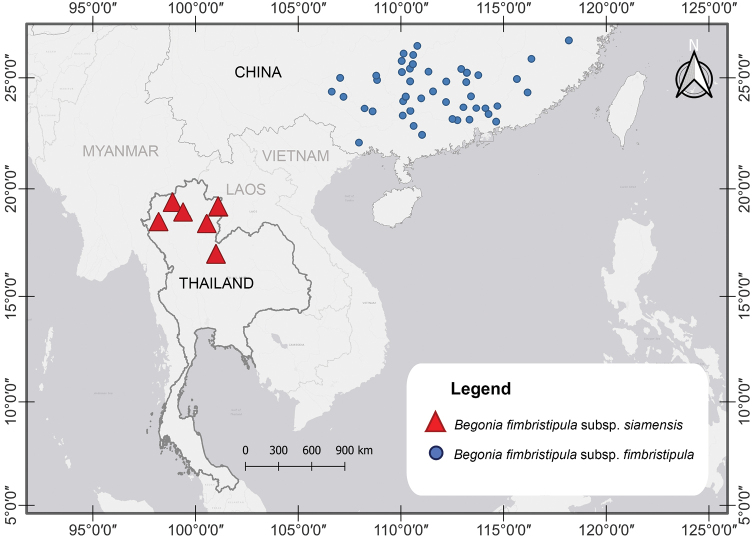
Distribution of B.fimbristipulaHancesubsp.siamensis Phutthai & S.Radbouchoom subsp. nov. (red triangle) and B.fimbristipulaHancesubsp.fimbristipula (blue circle).

The new taxon is currently known from seven collections obtained from three provinces located in northern and north-eastern Thailand (Fig. 2), where it grows on both limestone and sandstone outcrops in evergreen forest at high elevation (1300–1900 m). Individuals growing on limestone rocks are distinct from individuals growing on sandstone rocks by the presence of a dense indumentum formed by red trichomes. The indumentum of individuals growing on sandstone is less dense and the trichomes have a brighter colour. Accessions of Begoniafimbristipulasubsp.siamensis show high phenotypic variation among individuals of the same population especially in leaf size and abaxial leaf surface colour (Fig. 1H, H’). The notable phenotypic disparity resembles reports from occurrences of B.fimbristipulasubsp.fimbristipula in southern China (Wang et al. 2016; Tian et al. 2018). Phenotypic plasticity has been considered to result from adaption to the harsh environments occupied by these plants (Wang et al. 2016).

##### Phenology.

Flowering Time: June-September; Fruiting Time: August-December.

##### Etymology.

The subspecific epithet siamensis alludes to the nativity of this taxon to “Siam”, the exonym that was used for Thailand before 1949.

##### Conservation assessment.

The recorded occurrences of the new subspecies were mostly obtained in protected areas, e.g. National Parks. The high protection is arguably reduced by threats caused by tourism activities. In Phisanulok province, this species creates a beautiful landscape by growing on the sandstone cliff along the trekking trail, which is a famous spot among tourists. Some of its populations are outside the protected area where the new taxon has been threatened by illegal trade in wild ornamental plants, and habitat infringement. Until now, there is still no conservation plan for the new subspecies at any of these localities. Further surveys are arguably required to obtain a more accurate description of the distribution of this species in Thailand but also in Laos, Myanmar and Vietnam. The currently known area of occupancy (AOO) has been estimated as 32 km^2^ (Bachman et al. 2011; http://geocat.kew.org/). Based on IUCN criteria, the status assigned is “Vulnerable” (VU: B2 a, c (iii, iv); C2 (ai, ii)) (IUCN 2019).

##### Additional specimens examined.

**Thailand** – **Chiang Mai Prov.** • Pang Hin Fon, Mae Chaem District; 1300 m a.s.l.; 09 June 2017; W. Pongamornkul 6422; herb 20 cm high in dry evergreen forest, flowers pink; QBG 105946 • Dong Yen, Doi Chiang Dao; 1900 m a.s.l.; 20 Nov. 1999; P. Suksathan 2134; on mossy rock in shade; QBG16461. – **Phitsanulok Prov.** • Phu Hin Rong Kla national park; 1300–1600 m a.s.l.; 22 Jan. 2005; O. Kudjabnak, D. Watanachaiyingcharoen BRT (NU) 0046; herb on sand-stone; BKF SN152894. – **Nan Prov.** • Doi Phu Kha National Park, hill evergreen forest; 19°13'N, 101°06'E; 1750 m a.s.l.; 26 July 1999; P. Srisanga 918; herb on rock, leaves dark greenish-red, flower pink, stamen and stigma yellow; QBG15203 • ibid, Pua; 19°10'N, 101°07'E; 1800 m a.s.l.; 31 Aug. 2000; P. Srisanga 1533; hill evergreen forest, herb on rock, leaves greenish red, stem and pedicel red; QBG17980; BKF SN193126 • ibid, trail from Lan Doo Dao Phatang; 1700 m a.s.l.; 26 June 2008; R. Pooma, M. Tamura 7104; on moist rock surfaces, densely mosses, lower montane forest; BKF SN188535 • Doi Pha Phung, Nam Tok, Na Noi District; 18°24'22.06"N, 100°32'50.24"E; 992 m a.s.l.; 18 December 2018; P. Phaosrichai, M. Wongnak, S. Wongwan, S. Sitthisuk 1265; herb about 20 cm high, fruit brownish-red; QBG112488.

##### Specimens examined of BegoniafimbristipulaHancesubsp.fimbristipula.

**China** – **Fujian Prov.** • Wuyishan, on the way from ticketing entrance to Tienyoufeng; 270 m a.s.l.; 30 May 1997; Ching-I Peng 16885; HAST132046. – **Guangdong Prov.** • Ding-hu Shan; 06 May 1882; C. Ford 6; isolectotype K K000251078 • ibid; 150 m a.s.l.; 15 Apr. 1964; K.C. Ting, K.L. Shi 1402; BKF SN006485 • ibid; 6 May 1928; W.Y. Chun 6393; E E00299226 • ibid, Qingyuan Shi, Liannan Xian, Dalongshan Forest Plantation; 310 m a.s.l.; 4 Apr. 2005; Ching-I Peng 19496; on mossy rocky slope; HAST122379 • ibid; 26 May 1918; C.O. Levine 2027; E E00299227 • South of Ping Yung; 60–175 m a.s.l.; June 1924; R.C. Ching 2002; P P06844316. – **Guangxi Prov.** • Zhuangzu Zizhiqu, Laibin Shi, Jinxiou Yiaozu Zizhixian, Dayiaoshan national nature reserve; 1200 m a.s.l.; 18 September 2003; Wai-Chao Leong 3673; on mossy rock face, mixed Cathaya and broadleaf forest; HAST 97112.

### ﻿Key to the subspecies of *Begoniafimbristipula* Hance

**Table:** 

1	Tuber globose or sub-globose; petiole villous; peduncle glabrous; female tepals 3	**subsp. fimbristipula**
–	Tuber globose or sub-elongate; petiole densely red villous and pubescent; peduncle sparsely red villous; female tepals 5	**subsp. siamensis**

## ﻿Discussion

The new finding of Begoniafimbristipulasubsp.siamensis supports our expectation that several begonia species occurring in Thailand are still awaiting discovery. Our results are consistent with the accumulation of new species reported in recent studies, such as *B.exposita* Phutthai & M.Hughes, *B.fulgurata* C.I Peng, C.W.Lin & Phutthai, *B.pengchingii* Phutthai & M.Hughes, *B.pseudosubperfoliata* Phutthai & M.Hughes, *B.phutthaii* M.Hughes, *B.sirindhorniana* Phutthai, Thananth., Srisom & Suddee, *B.tenasserimensis* Phutthai & M.Hughes (Peng et al. 2017; Phutthai and Hughes 2017a, 2017b; Phutthai et al. 2019, 2021). In this study, we report a new subspecies of Begoniasect.Diploclinium from Thailand, resulting in the number of Begoniasect.Diploclinium species known from Thailand increasing to 20 species (of which one is an endemic subspecies). We treat the newly found taxon as subspecies B.fimbristipulasubsp.siamensis because of the distinct morphological affinity to B.fimbristipulasubsp.fimbristipula. A comparison between these two taxa and a third similar species, namely *B.poilanei* Kiew (Kiew 2007; Shui and Chen 2017), was assembled (Table 1). Our taxonomic conclusions consider also the geographic isolation of the new taxon occurring only in northern and north-eastern Thailand (Chiangmai, Nan, Phitsanulok,) away from typical *B.fimbristipula* occurring exclusively in southern China (Fujian, Guangdong, Guangxi, Hainan, Hunan, Jiangxi, Zhejiang). Thus, they are geographically disjunct taxa but future studies need to confirm the absence of occurrences bridging the gap in eastern Myanmar, northern Laos and northern Vietnam. In this context, it is worth noting that several putative relatives based on shared morphological similarities are known to occur in this region including *B.poilanei* from China and Vietnam (Kiew 2007) and *Begoniamengdongensis* H.H.Xi from southeastern Yunnan, China (Xi et al. 2020). Currently, it is also not known if the Thailand accession may also be harvested as a natural resource similar to the typical *B.fimbristipula*, whose plants are used to brew herbaceous teas, and as herbal medicine (Zhao et al. 2016).

## Supplementary Material

XML Treatment for Begonia fimbristipula Hance subsp. siamensis
